# A Love Wave Reflective Delay Line with Polymer Guiding Layer for Wireless Sensor Application

**DOI:** 10.3390/s8127917

**Published:** 2008-12-05

**Authors:** Wen Wang, Shitang He

**Affiliations:** Institute of Acoustics, Chinese Academy of Science, Beijing, 100190, P.R. China

**Keywords:** Reflective delay line, Love wave, PMMA, 41^°^ YX LiNbO_3_, COM

## Abstract

This paper presents an optimal design for a Love wave reflective delay line on 41^°^ YX LiNbO_3_ with a polymer guiding layer for wireless sensor applications. A theoretical model was established to describe the Love wave propagation along the larger piezoelectric substrate with polymer waveguide, and the lossy mechanism from the viscoelastic waveguide was discussed, which results in the optimal guiding layer thickness. Coupling of modes (COM) was used to determine the optimal design parameters of the reflective delay line structured by single phase unidirectional transducers (SPUDTs) and shorted grating reflectors. Using the network analyzer, the fabricated Love wave reflective delay line was characterized, high signal noise ratio (S/N), sharp reflection peaks, and few spurious noise between the peaks were found, and the measured result agrees well with the simulated one. Also, the optimal guiding layer thickness of 1.5∼1.8µm was extracted experimentally, and it is consistent with the theoretical analysis.

## 1. Introduction

**Figure 1.**
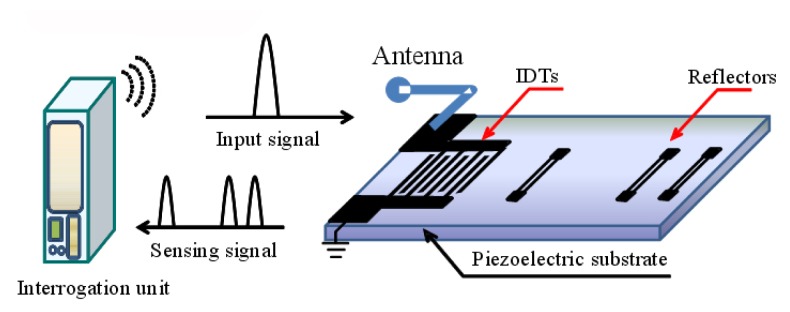
The working principle of the wireless sensor based on SAW reflective delay line.

Love devices are used widely for bio(chemical) sensor applications because these devices provide low acoustic loss in contact with liquid, high sensitivity, and good protection of the interdigital transducers (IDTs) from harsh gas and liquid environments [1-4]. Love wave is shear horizontal (SH) polarized guided waves, propagating in a layered structure consisting of a substrate with high shear acoustic velocity and a waveguide layer with a low shear acoustic velocity on top of it. Because of the shear nature of the wave, the devices can operate in the presence of liquid without any losses occurring due to the mode conversion. Of significance, when properly optimized, the acoustic energy is confined to the sensing surface resulting in high sensitivity to surface perturbation as mass loading. A great variety of Love wave sensors with different designs and geometries were reported. A typical Love wave sensor was composed of delay lines with oscillation configuration, which provide high resolution of frequency signals and good temperature compensation for external temperature changes. However, due to the active oscillator structure, the Love wave sensor structured by delay line patterns cannot be used in wireless sensor applications for some toxic and dangerous environments because of the power system requirements. Recently, use of a SAW reflective delay line pattern as the wireless gas sensor element with some meaningful results as high sensitivity, passive absolutely was reported [5]. It was composed of interdigital transducers (IDTs) and several reflectors positioned on the piezoelectric substrate in a row. The reflectors are located at different distances from IDTs to get different wave propagation lengths. The working principle of the wireless sensor based on SAW reflective delay line is shown in Figure 1. When the IDTs of the SAW reflective delay line receive electromagnetic (EM) energy from interrogation unit through antennas, the SAW is generated on the substrate and propagating toward reflectors. The propagating SAW partially reflects from the reflectors, reconverted into EM waves by the IDTs and transmitted to the interrogation unit. The external perturbation as mass loading effect results in the linearly phase shifts of the reflection peaks, which was used to evaluate the target species. Till the present, to our knowledge, there have been few reports concerning wireless sensor applications based on Love wave reflective delay line, which is structured by a SH-SAW reflective delay line and a guiding layer coated on top of it. Due to the waveguide effect, it is promising for the mass loading sensitivity improvement of the bio(chemical) sensor. Superior sensor performance require the Love wave reflective delay line with high S/N, few spurious noise and sharp reflection peaks, and which are determined by the optimal guiding layer thickness and design parameters of the SH-SAW reflective delay line.

**Figure 2.**
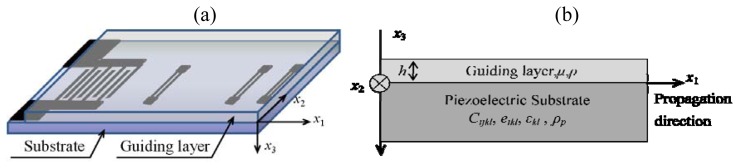
(a) The schematic of the Love wave reflective delay line. (b) The system coordinates of the theoretical model.

The first purpose of this paper is to describe the Love wave propagation along a substrate with larger piezoelectricity (41^°^ YX LiNbO_3_) with a polymer guiding layer (PMMA: stiffness modulus of 1.7 GPa and a density of 1.17 g/cm^3^, giving a lower waveguide acoustic velocity of 1105 m/s [6]) on top of it, as shown in Figure 2 (a). A theoretical model of a Love wave propagating in a viscoelastic polymer layer deposited on a piezoelectric substrate was considered, and an analytical formula relating the attenuation coefficient of the Love wave and the viscoelastic parameters of the waveguide structure were established. The complex dispersion relationship of the Love wave was studied, in which, the attenuation induced by the viscoelastic guiding layer was calculated, and it would provide as guidelines for the optimization structure of the Love wave devices.

Another aim of this paper was to determine the optimal design parameters of the SH SAW reflective delay line on 41^°^ YX LiNbO_3_ by COM models. Based on the simulated results, a 440 MHz Love wave reflective delay line with SPUDTs and three shorted grating reflectors was fabricated by standard photographic techniques. 41^°^ YX LiNbO_3_ was used for the piezoelectric substrate because it supports a SH SAW with a relatively high SH velocity and large electromechanical coupling coefficient *K*^2^ [7]. The basic idea of the SPUDT is to enhance the generated signal in the forward direction but reduce the signal in the reverse direction using the distributed reflection sources (λ/4 reflection electrode), which suppress triple transit and reduce insertion loss effectively [8]. Various thicknesses of PMMA are deposited onto the substrate to extract the optimal guiding layer thickness. Experimental data was consistent with the theoretical analysis.

## 2. Theoretical analysis

### 2.1. Love wave model

For the theoretical approach, the Love wave device can be considered as a multilayer composed of a semi-infinite piezoelectric substrate and a guiding layer. The piezoelectric substrate acts as a mechanical support and allows generating elastic waves by IDTs. For Love wave in isotropic structure, solution was given by Royer *et al.* [9]. In addition, Zimmermann *et al.* presented the weak piezoelectric Love wave structure like quartz [10]. Wang *et al.* described the Love wave propagation along the multi-layered structure as PMMA/SiO_2_/LiNbO_3_ [11].

Kielczynski established the theory of Love waves propagating in a lossy viscoelastic layer deposited on an elastic substrate, in which the attenuation induced by the lossy guiding layer was described numerically [12]. In this paper, we propose a theoretical propagation solution for a Love wave in a multilayer structure composed of an anisotropic substrate with large piezoelectricity (example of 41^°^ YX LiNbO_3_ with Eular angles of (0, -49, 0)), and a isotropic guiding layer using the method descrbed in [11]. The coordinate system for the Love wave propagation analysis is shown in Figure 2(b). Acoustic wave propagates along the *x*_1_ axis on the *x*_1_-*x*_2_ plane at *x*_3_=0. The attenuation coefficient of the PMMA guiding layer on Love wave propagation is calculated approximately referring the numerical method of [12].

A necessary condition for obtaining Love wave propagation in the guiding layer is shear horizontal polarization. Symmetry properties in structures considered in this paper, allow the reduction of the equations of motion to a single displacement equation and electrical potential for the substrate:

(1) $$\begin{array}{l} \rho_{s}\partial^{2}u_{2}/\partial t^{2}=C_{66}\partial^{2}u_{2}/\partial {x_{3}}^{2}+C_{44}\partial^{2}u_{2}/\partial {x_{3}}^{2}+e_{16}\partial^{2}\phi /\partial {x_{1}}^{2}+e_{34}\partial^{2}\phi /\partial {x_{3}}^{2}\\ e_{16}\partial^{2}u_{2}/\partial {x_{1}}^{2}+e_{34}\partial^{2}u_{2}/\partial {x_{3}}^{2}=\varepsilon_{11}\partial^{2}\phi /\partial {x_{1}}^{2}+\varepsilon_{33}\partial^{2}\phi /\partial {x_{3}}^{2} \end{array}$$

Here *u_2_* is the acoustic displacement of particles in *x_2_* direction, *C_44_*, and *C_66_* are subscript abbreviation of stiffness constants, *e_16_* is the piezoelectric modules, *ε_33_* is the component of permittivity, and *ρ_s_* is the density of the considered layer. The displacement motion equation in waveguide layer can be described as the following, with shear modulus *G_g_* and density *ρ_g_* of guiding layer and sensitive film:

(2) $$G_{g}\partial^{2}u_{2}/\partial x_{3}+(\rho_{g}\omega^{2}-k^{2}G_{g})u_{2}=0.$$

Based on the mechanical boundary conditions (zero stress at the top of the structure, continuity of stress and mechanical displacement at interfaces between the adjacent layers) and electrical (electric displacement continuity at the interface between the substrate and guiding layer), the dispersion relation describing the Love wave propagation in the LiNbO_3_/PMMA can be written by:

(3) $$\begin{array}{l} h=1/(k\alpha_{g})\times \arctan(-i(\lambda_{1}\lambda_{4}+\lambda_{2}\lambda_{3})/(\lambda_{1}+\lambda_{2})G_{g}\alpha_{g})+n\pi /(k\alpha_{g})\\ \alpha_{g}=\sqrt{\rho_{g}\nu_{\text{Love}}/G_{g}-1}\\ \lambda_{1}=i\varepsilon_{33}\alpha_{p1}k_{1}-ie_{16}\alpha_{p1}-\varepsilon_{0}k_{1},\lambda_{2}=\varepsilon_{0}k_{2}+ie_{16}\alpha_{p2}-i\varepsilon_{33}\alpha_{p2}k_{2}\\ \lambda_{3}=C_{44}\alpha_{p1}+e_{16}k_{1}\alpha_{p1},\lambda_{4}=C_{44}\alpha_{p2}+e_{16}k_{2}\alpha_{p2}\\ \lambda_{5}=G_{s}\alpha_{s}\tan(\alpha_{s}kh_{s}),k_{1}=(\rho {\nu_{\text{Love}}}^{2}-C_{66}-C_{44}{\alpha_{p1}}^{2})/(e_{16}+e_{16}{\alpha_{p1}}^{2})\\ k_{2}=(\rho \nu^{2}-C_{66}-C_{44}{\alpha_{p2}}^{2})/(e_{16}+e_{16}{\alpha_{p2}}^{2}) \end{array}$$

where *n* is an integer which represents mode order, *a_p_*_1_ and *a_p_*_2_ are decay constants for LiNbO_3_, *v_Love_* is the Love wave velocity, and *h_g_* and *h_s_* are thicknesses of guiding layer and sensitive layer.

Owing to the viscoelastic nature of the polymer materials, the attenuation induced by the polymer guiding layer on wave propagation should be taken into account. In this paper, we assume that the dispersion relationship of the Love wave propagation is complex with the complex wave vector *k* (*k*=*k_0_*+*jα*, the real part of the wave vector *k_0_* describes the phase velocity of the Love wave and the imaginary part of the wave vector *α* is an attenuation coefficient of the Love wave induced by the polymer guiding layer) and complex shear modulus of the guiding layer *G_g_* (*G*=*G′*+*jG″*, *G′* is the storage shear modulus of the PMMA and *G″* represents the loss module). To simplify the numerical calculation, we neglect the piezoelectric properties of the LiNbO_3_ substrate. Through the Taylor series expansion mentioned in the Reference 11, the attenuation coefficient of the PMMA with thickness of *h* on the wave propagation can be obtained as the following:

(4) αh=−G″G'×12cos2(Ah)ρg.ν2G'×(k0h)2+tan(Ah)[12Ahρg.ν2(k0h)2G'−Ah]k0hcos2(Ah)+ρsC44k0hBG'h(C44C66−C46)+tan(Ah).k0hAhA=ρgw2/G'−k02,B=k02−w2ρsC44/(C44C66−C46)

In this section, we illustrate the numerical results of fundamental properties of the love wave from a layered structure of PMMA/LiNbO_3_, dispersion relation. The stiffness constants, piezoelectric modules and permittivity constants of LiNbO_3_ are obtained from [13]. These were used in the numerical calculations presented below.

### 2.2. Dispersion relation

**Figure 3.**
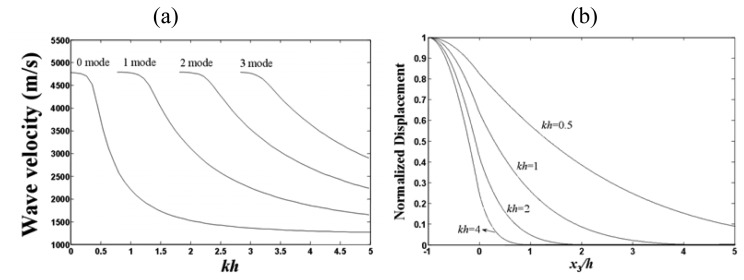
(a) phase velocity versus normalized waveguide layer thickness for first five modes in a 41^°^-YX LiNbO_3_-PMMA layered structure. (b) Love wave normalized displacement *u*_2_ versus normalized depth *x*_3_/*h* for different thickness of guiding layer.

As shown in Equation (3), the Love wave with multilayered structure is dispersed and multimode, which is different from other wave modes existing in semi-infinite substrate. Figure 3(a) shows the phase velocity as a function of normalized layer thickness *kh* (*k*: wavenumber depending on the operation frequency and *h*: PMMA thickness) for the fundamental mode and the next three Love wave modes in a 41^°^ YX LiNbO_3_ with a PMMA guiding layer. In the case of the fundamental mode with very thin PMMA layer, most of the acoustic energy propagates in the substrate and, consequently, the phase velocity is close to the shear velocity of LiNbO_3_. With increasing waveguide layer thickness, the wave velocity is decreased, and for very thick layers it approaches the shear velocity of PMMA. Moreover, the number of love wave modes is decreased even if only one mode exists in the thin guiding layer. Figure 3(b) shows a schematic representation of the variation in the love wave particle displacement *u_2_* as a function of overlayer thickness (*x*_3_/*h*) for the first mode of operation. With a low thickness of the guiding layer, the acoustic field deeply penetrates into the substrate (*kh*<0.5) and the love wave propagates with a velocity, *v_Love_*, that is very close to shear velocity of substrate *v_substrate_*. With increasing thickness, the guiding becomes more efficient (*kh*=0.5∼2), the penetration depth decreases, a larger fraction of the total wave energy propagates in the overlayer, and the *v_love_* tends toward to shear velocity of guiding layer *v_layer_*. For very thick layers (*kh*>2), almost the entire energy is trapped into the guiding layer and the love wave velocity is close to the *v_layer_*. Between these two limits (*kh*<0.5 and *kh*>2), the energy progressively transits from the LiNbO_3_ substrate into the layer and the velocity *v_Love_* varies between *v_substrate_* and *v_layer_*, that is, *v_layer_* < *v_Love_* < *v_substrate_*. Finally, in addition to the energy distribution, the particle displacement on the surface of the PMMA layer increases with increasing overlayer thickness, which results in structures that are more sensitive to surface perturbations.

**Figure 4.**
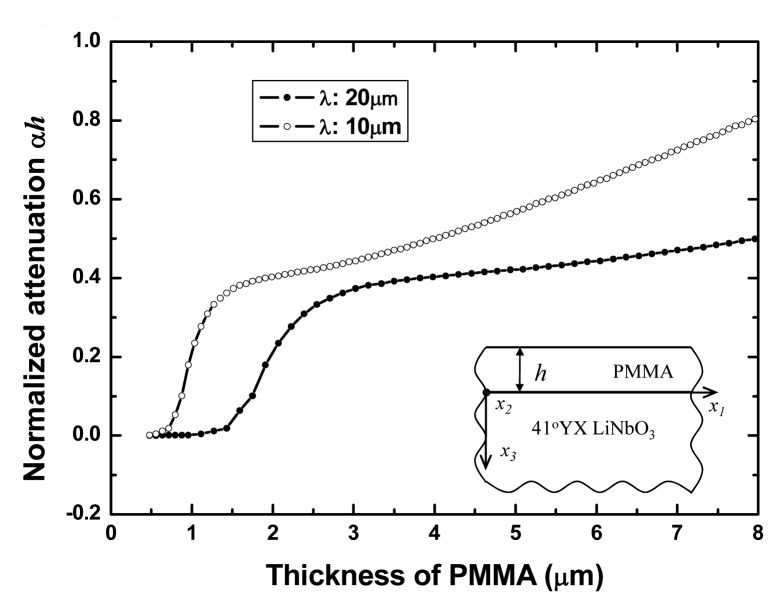
The attenuation coefficient versus PMMA guiding layer thickness as a function of different operation frequency.

However, as the PMMA thickness increases, the attenuation in the PMMA becomes the predominant mechanism of acoustic loss due to the viscoelatic property of polymer film, so it can not be ignored, as shown in Figure 4. In this simulation, the complex shear modulus of the PMMA is assumed as 1.7e^10^+*j*×1.4e^8^*Pa*.Figure 4 shows the dispersion curves of the attenuation coefficient α*h* versus PMMA thickness *h* employing Equation 4 at different operation frequency (represented by various wavelength, 10λ and 20λ). From the picture, the *αh* increases monotonically with the layer thickness *h*, also, large attenuation coefficient *αh* is observed at higher operation frequency (10λ). The calculated result shows that the thick PMMA guiding layer will induce large acoustic attenuation of the Love wave devices. This suggests that the lossy nature of the polymer film determine the optimum guiding layer thickness of the Love devices and should be taken into account when designing an efficient Love wave device and the suitable thickness of the PMMA guiding layer can be considered small than 3µm in our work.

## 3. COM Analysis

COM modeling is a very efficient technique developed for the analysis of the SAW device in which small distributed internal reflections are important, such as SPUDT structure. COM model provides an efficient and highly flexible approach for modeling various kinds of SAW devices [14]. For the SAW reflective delay line, COM model was used to analyze the IDT (SPUDT used in this paper) and reflectors respectively. Then, using the so called mixed *P*-matrix and FFT program, the reflective coefficient S_11_ in time domain of the SAW device was deduced.

### 3.1. COM analysis for SPUDT

**Figure 5.**
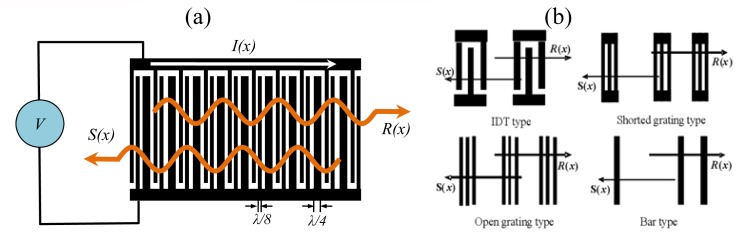
(a) SAW schematic and variables for COM theory of the IDTs. (b) and Reflectors for the SAW reflective delay line.

Wright deduced the COM equation for SAW devices with SPUDT [15]. The COM equation deals with acoustic waves propagating in the forward and reverse directions and incorporates their coupling interaction, as shown in Figure 5(a). *R(x)* and *S(x)* are two slowly varying acoustic wave amplitudes. The 3×3 *P*-matrix representation is used to present the solutions to the COM equations [Equation (1)] [16]. The *L* is transducer length. The three equations in the COM modeling can be integrated, so that all parameters in the *P*-matrix can be evaluated as the following.

(5) $$\left[\begin{array}{c} S(0)\\ R(L)\\ I \end{array}\right]=\left[\begin{array}{ccc} P_{\text{IDT}11} & P_{\text{IDT}12} & P_{\text{IDT}13}\\ P_{\text{IDT}21} & P_{\text{IDT}22} & P_{\text{IDT}23}\\ P_{\text{IDT}31} & P_{\text{IDT}32} & P_{\text{IDT}33} \end{array}\right]\;\left[\begin{array}{c} R(0)\\ S(L)\\ V \end{array}\right]$$

### 3.2. COM analysis for reflectors

Figure 5(b) shows the configuration of various type reflectors. The COM analysis of the IDT type reflectors was mentioned above, whereas for the shorted circuit grating reflector, as shown in Figure 5 (b), the COM equation is presented by

(6) $$\{\begin{array}{cc} \frac{\text{dR}(x)}{\text{dx}} & =-i\delta R(x)+i\kappa S(x)\\ \frac{\text{dS}(x)}{\text{dx}} & =-i\kappa^{*}R(x)+i\delta S(x) \end{array}$$

where the δ is the detuning factors, and *κ* is the reflectivity. The 2×2 mixed P-matrix was used to present the solutions to the Equation (6).

(7) $$\left[\begin{array}{c} S(0)\\ R(L) \end{array}\right]=\left[\begin{array}{cc} P_{\text{ref}11} & P_{\text{ref}12}\\ P_{\text{ref}21} & P_{\text{ref}22} \end{array}\right]\;\left[\begin{array}{c} R(0)\\ S(L) \end{array}\right]$$

Then, the COM equation for open circuit grating reflector is:

(8) $$\{\begin{array}{cc} \frac{\text{dR}(x)}{\text{dx}} & =-i\delta_{\text{oc}}R(x)+i\kappa_{\text{oc}}S(x)\\ \frac{\text{dS}(x)}{\text{dx}} & =-i{\kappa_{\text{oc}}}^{*}R(x)+i\delta_{\text{oc}}S(x) \end{array}$$

where *δ_oc_*=*δ*-2|*α*|^2^/(*ωC*), *κ_oc_*=*κ*+2*α*/(*ωC*) [14]. Similar to the shorted circuit reflectors, the open circuit grating reflectors can also be described as the 2×2 mixed *P*-matrix.

As for the bar type reflector, it can be regard as a special open circuit grating reflector with unit length of ¼λ (only a finger for one reflector), where λ corresponding to the operate frequency.

### 3.3. Reflection coefficient S_11_ of the reflective delay line

Using the cascading relationships [16], the *P*-matrix for all the individual IDT segments and the transmission matrix between the IDT and first reflector can be cascaded and described as *P_TIDT_*. The *P* matrix for the reflectors is also cascaded as *P_TReference_* Therefore the admittance matrix for the whole device can be expressed by

(9) $$Y=\left[\begin{array}{cc} y_{11} & y_{12}\\ y_{21} & y_{22} \end{array}\right]$$

where

$$\begin{array}{cccc} y_{11}=P_{\text{TIDT}33}+\frac{{\text{P}}_{\text{Tref}11}P_{\text{TIDT}32}P_{\text{TIDT}23}}{1-P_{\text{Tref}11}P_{\text{TIDT}22}}, & y_{12}=\frac{{\text{P}}_{\text{Tref}13}P_{\text{TIDT}32}}{1-P_{\text{Tref}11}P_{\text{TIDT}22}}, & y_{21}=\frac{P_{\text{Tref}31}P_{\text{TIDT}23}}{1-P_{\text{Tref}11}P_{\text{TIDT}22}}, & y_{22}=P_{\text{Tref}33}+\frac{P_{\text{TIDT}22}P_{\text{Tref}13}P_{\text{Tref}31}}{1-P_{\text{Tref}11}P_{\text{TIDT}22}} \end{array}$$

Using the admittance matrix solution, the reflection coefficient S_11_ can be deduced by

(10) $$S_{11}=\frac{(Y_{G}-y_{11})\times (Y_{G}+y_{22})+y_{12}\times y_{21}}{(Y_{G}+y_{11})\times (Y_{G}+y_{22})-y_{12}\times y_{21}}$$

where, *Y_G_* is the resource and load inductance. Using the FFT program, the S_11_ in frequency domain can be transfer into time domain.

**Figure 6.**
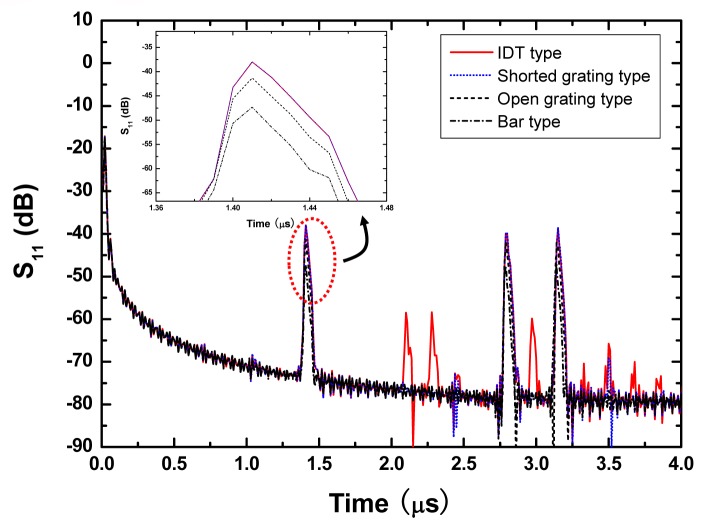
Simulated S_11_ of the reflective delay line with SPUDTs and various types of reflectors based on COM model.

Then, a 440 MHz SH SAW reflective delay line with SPUDT and three various type reflectors is simulated. A 41^°^ YX LiNbO_3_ piezoelectric substrate was used. Figure 6 shows the simulated reflection coefficient S_11_ in time domain in case of 41^°^ YX LiNbO_3_, aluminum SPUDT with 20 finger pairs, 100 λ aperture size (λ means the wavelength corresponding to the operation frequency) and three various type reflectors (IDT, shorted grating, open grating and bar types). Other parameters used in COM simulation are listed in Table.1. Among several different reflector structures, shorted grating reflector can reduce the spurious signals between the reflection peaks, and decrease the insertion loss effectively due to the zero self reflection and strong reflectivity of the reflector.

## 4. Technique Realization

### 4.1. SH SAW reflective delay line

**Figure 7.**
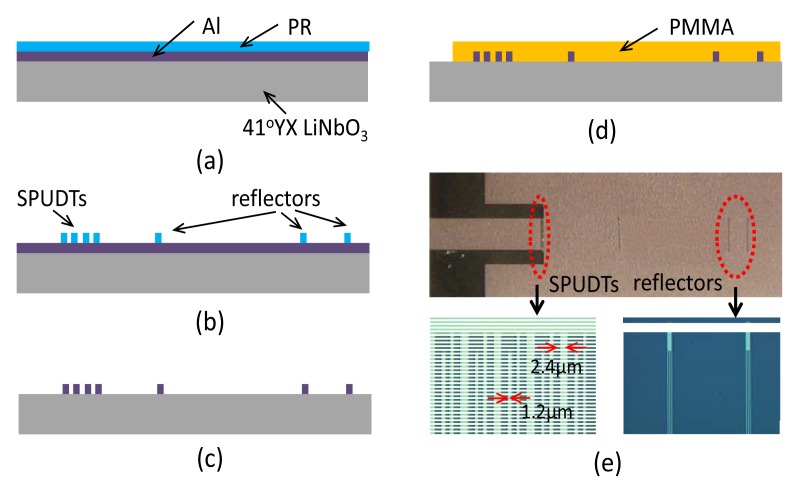
Fabrication procedure of the Love wave reflective delay line (a) Al and PR coating, (b) development, (c) Al etching, (d) PMMA spin-coating, and (e) optical image of the fabricated Love wave device.

Based on the simulated results, a 440 MHz SH SAW reflective delay line on 41^°^ YX LiNbO_3_ with SPUDTs and three shorted grating reflectors was fabricated by standard photographic technique. Usually, the time interval between the SPUDTs and the first reflector was set to 1.2 µs to allow adequate separation between environmental noise echoes and reflection peaks, because all of the environment echoes fade away within ∼1 µs. to hold the uniformity between peaks, different electrodes are used to structure the shorted grating reflectors. The first reflector includes 3 electrodes, the second and third reflector include 5 and 6 electrodes reflectively. Figure 7 shows schematic diagrams of the fabrication procedure. A 4″ 41^°^ YX LiNbO_3_ with 500 µm thickness was used for the piezoelectric substrate. A ∼150 nm thick aluminum was deposited on the piezoelectric substrate using an electron beam evaporator. Photoresist (PR) was spin-coated, exposed and then patterned for IDT and reflectors [Figures 7 (a-b)]. The aluminum was wet-etched [Figure 7 (c)]. PR was dissolved in acetone. Several rinses with DI water were performed to remove any unwanted products.

### 4.2. PMMA waveguide layer

From the theoretical simulation works, the PMMA thickness should be smaller than 3 μm to avoid the larger viscoelastic acoustic attenuation. To extract the optimal thickness of PMMA guiding layer, a layer of polymethylmethacrylate (PMMA) with various thicknesses was deposited on the entire fresh SH SAW reflective delay line surface expects for electrical connected pads by spin coating solution of 8-9% PMMA (MW: 495,000) in chlorobenzene, obtained from Micro-Chem, at 1,200 r/min for 300 s, and then cross-linked by heating the device for 2 h at 180°C (Figure 7 (d)). Thickness was targeted from 0.3 µm to 2 µm, and monitored by the talysurf profilometer.

Figure 7(e) shows optical microscope views of the fabricated devices. The SPUDT finger pair was 20, and the finger widths were ∼1.244 mm and 2.488 mm, respectively. The length of the aperture was 100 λ (∼1mm). Three shorted grating reflectors were arranged in a row on the 41^°^ YX LiNbO_3_ substrate. The distance between the SPUDT and the first reflector was 2.672 mm, and the distance between the first and the second reflector was 3.95 mm. PMMA thickness was varied from 0.3µm to 2.5 µm to find an optimized waveguide layer thickness. Uniform waveguide surface was obtained over the delay line regions.

## 5. Results and Discussion

### 5.1. Electric measurement

**Figure 8.**
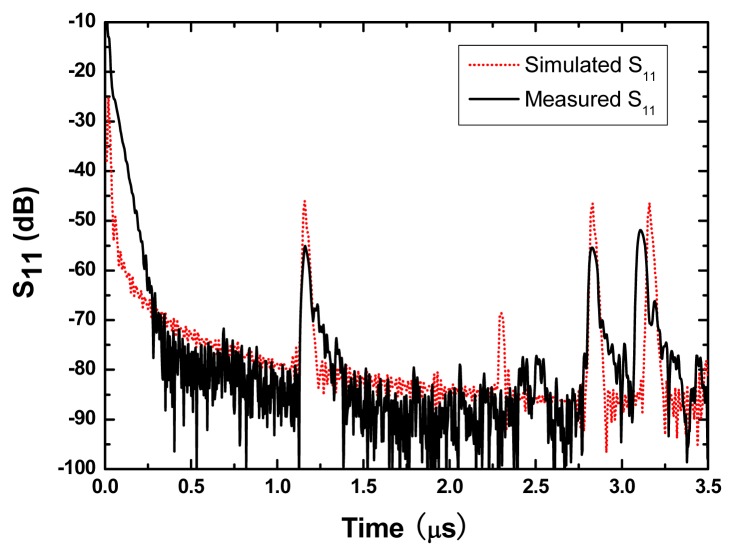
Comparison between the measured S_11_ and simulated S_11_ of SH SAW reflective delay line.

The fabricated SH SAW device was characterized by the network analyzer. As shown in Figure 8, three reflection peaks from three reflectors were observed in the time domain. The peaks showed large S/N ratio, sharp peaks, better uniformity between peaks and few spurious signals. The first reflection peak occurred at 1.2 μs, and at that point, S_11_ was ∼55 dB. Also, the measured result agrees well with the simulated one. From the measured results, we can found that (1) the structure of SPUDTs can decrease the insertion loss and improve the S/N; (2) the spurious noise between the reflections peaks can be reduced by the shorted grating reflector effectively; (3) the appropriate arrangement of the reflectors with different length can hold equal intensities for all the reflection peaks. The length of the reflectors should be short in near from IDT and long in far from IDT.

### 5.2. Waveguide thickness effect

**Figure 9.**
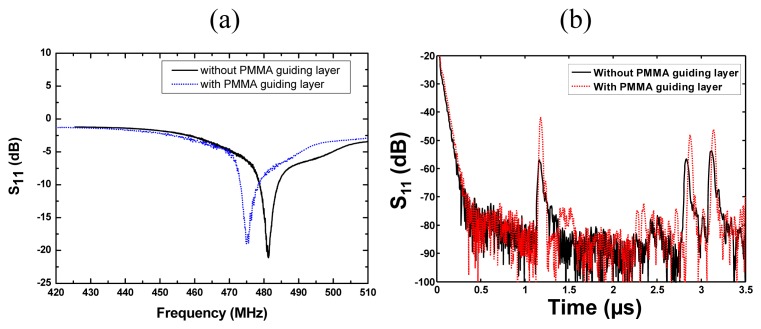
Frequency shift (a) and amplitude change (b) of the SH SAW reflective delay line coated with PMMA guiding layer.

Different waveguide thicknesses were spin-coated on the piezoelectric substrate and the efficiency of each waveguide was compared by monitoring the resonant frequency and insertion loss of the device. Figure 9 shows the frequency shift and amplitude change of the reflective delay line with 1.5 µm PMMA guiding layer. Due to the mass loading from PMMA deposition, the SH-SAW mode was converted into Love wave mode with decrease of the wave velocity, which results in the frequency change in Figure 9 (a). Also, the loss of the reflection peaks in time domain was decreased to over 10 dB owing to the waveguide effect of PMMA coating.

**Figure 10.**
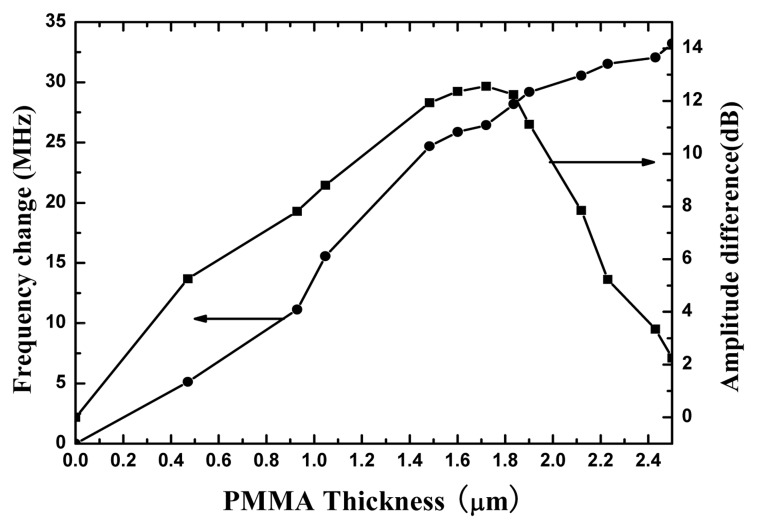
The changes of the resonance frequency and the amplitude of the reflection coefficient S_11_ as a function of PMMA thickness.

Then, the effect of the polymer layer thickness on the acoustic response of the device was studied. Various devices with PMMA layer thicknesses range of 0.3∼2.5 μm have been initially characterized. The velocity change, Δ*v* as a function of the PMMA layers thickness for fundamental Love wave mode is shown in Figure 10, which is obtained from frequency shift Δ*f* of S_11_ in frequency domain through Δ*f*/*f*_0_≈ Δ*v*/*v*_0_, monitored by the network analyzer. Love wave velocity decreases with the increasing of polymer layers thickness, which is in accordance with theoretical predictions [Figure 3(a)]. The loss (S_11_ in time domain) as a function of waveguide thickness is defined as the difference between the loss of the Love wave and uncoated device. The positive amplitude change indicates that the waveguide devices are less loss than the leaky SH SAW device because more acoustic energy is guided to the overlayer and the upper part of the piezoelectric substrate, which agrees to the Figure 3(b). Furthermore, Figure 10 shows amplitude change increases sharply with increasing the PMMA thickness for a thickness up to ∼1.2 μm and then reaches a plateau for thicknesses between 1 and 1.8 μm. For polymer layers over 1.8 μm thick, the amplitude change decreases fast, and for thicker layers, amplitude becomes considerably larger than that of the uncoated device. This can be explained by taking into account acoustic losses inside the polymer layer (viscoelastic properties), which become significant when thicker waveguide layers are applied on the device surface, and it is consistent with the Figure 5. Whereas for the thinner polymer layer, the acoustic energy can not be trapped the sensing surface efficiently. Thus, from the measured results, the optimum waveguide thickness is considered as 1.2∼1.8 μm, which agrees well with the simulated results (Figure 5).

## 6. Conclusion

This paper presents an optimal design for a Love wave reflective delay line with a polymer guiding layer for wireless sensor applications. Theoretical modes was used to describe the Love wave propagation along the structure of PMMA/41^°^YX LiNbO_3_, including the dispersion relationship and attenuation coefficient from the viscoelastic polymer guiding layer. COM model was used to determine the optimal design parameters of the SH-SAW reflective delay line. Using the network analyzer, the fabricated 440 MHz Love wave reflective delay line structured by SPUDT and shorted grating reflectors was characterized, high S/N, sharp peaks, better uniformity between peaks and few spurious signals were obtained, and measured result agrees well with the simulated result. Various thicknesses of PMMA were deposited onto the LiNbO_3_ substrate to extract the optimal guiding layer thickness of 1.5∼1.8 µm, which is consistent with the theoretical analysis.
